# The efficacy of 5.5-mm diameter rods combined with cervical pedicle screws for the treatment of challenging spinal disease in cervicothoracic junction: Is it a game-changer?

**DOI:** 10.1097/MD.0000000000044369

**Published:** 2025-09-05

**Authors:** Younggyu Oh, Subum Lee, Sang Hyub Lee, Danbi Park, Chongman Kim, Sun Woo Jang, Jin Hoon Park

**Affiliations:** a Department of Neurosurgery , Korea University Anam Hospital, Korea University College of Medicine, Seoul, Republic of Korea; b Department of Neurosurgery, Spine Center, The Leon Wiltse Memorial Hospital, Suwon, Republic of Korea; c Department of Neurological Surgery, Asan Medical Center, University of Ulsan College of Medicine, Seoul, Republic of Korea; d College of Nursing, Korea University, Seoul, Republic of Korea; e Department of Industrial and Management Engineering, Myongji University, Yongin, Republic of Korea; f Department of Neurological Surgery, Gangneung Asan Hospital, University of Ulsan College of Medicine, Gangneung, Republic of Korea.

**Keywords:** cervical pedicle screw, cervical spinal deformity, cervical spinal trauma, cervical spinal tumor, cervicothoracic junction, CPS, CTJ, short-segment fusion

## Abstract

The cervicothoracic junction (CTJ) presents a surgical challenge due to its transitional nature from mobile to rigid segments. Therefore, the biomechanical characteristics of this transitional zone must be taken into consideration during instrumentation. This study aimed to determine the efficacy of the cervical pedicle screw placement (CPS) combined with 5.5-mm single-diameter rods in treating various challenging diseases at the CTJ. From March 2018 to February 2022, a total of 42 patients (male = 25; female = 17) underwent posterior cervical spinal surgery crossing the CTJ with or without an anterior approach by a single surgeon. The mean patient age was 58.6 years (range, 16–81 years). In all the included cohorts, only the Legacy^®^ pedicle screw system (Medtronic Sofamor Danek, Inc., Memphis, TN, USA) was used for the placement of pedicle screws on the cervical and thoracic vertebrae at the CTJ. This study describes the detailed indications and results of the treatment for 5 disease categories. CPS combined with 5.5-mm single-diameter rods provides biomechanical stability for the treatment of challenging conditions, such as trauma, benign or malignant tumors, or deformities in the CTJ.

## 1. Introduction

The cervical thoracic junction (CTJ) is a challenging anatomical transitional area for the treatment of spinal diseases.^[1–5]^ Despite several controversies, the consensus on surgical treatment has shown a consistent trend toward obtaining stronger biomechanical stability.^[4]^ When instrumentation crosses the CTJ, the biomechanical characteristics of this transitional zone must be taken into consideration.

**Figure 1.**
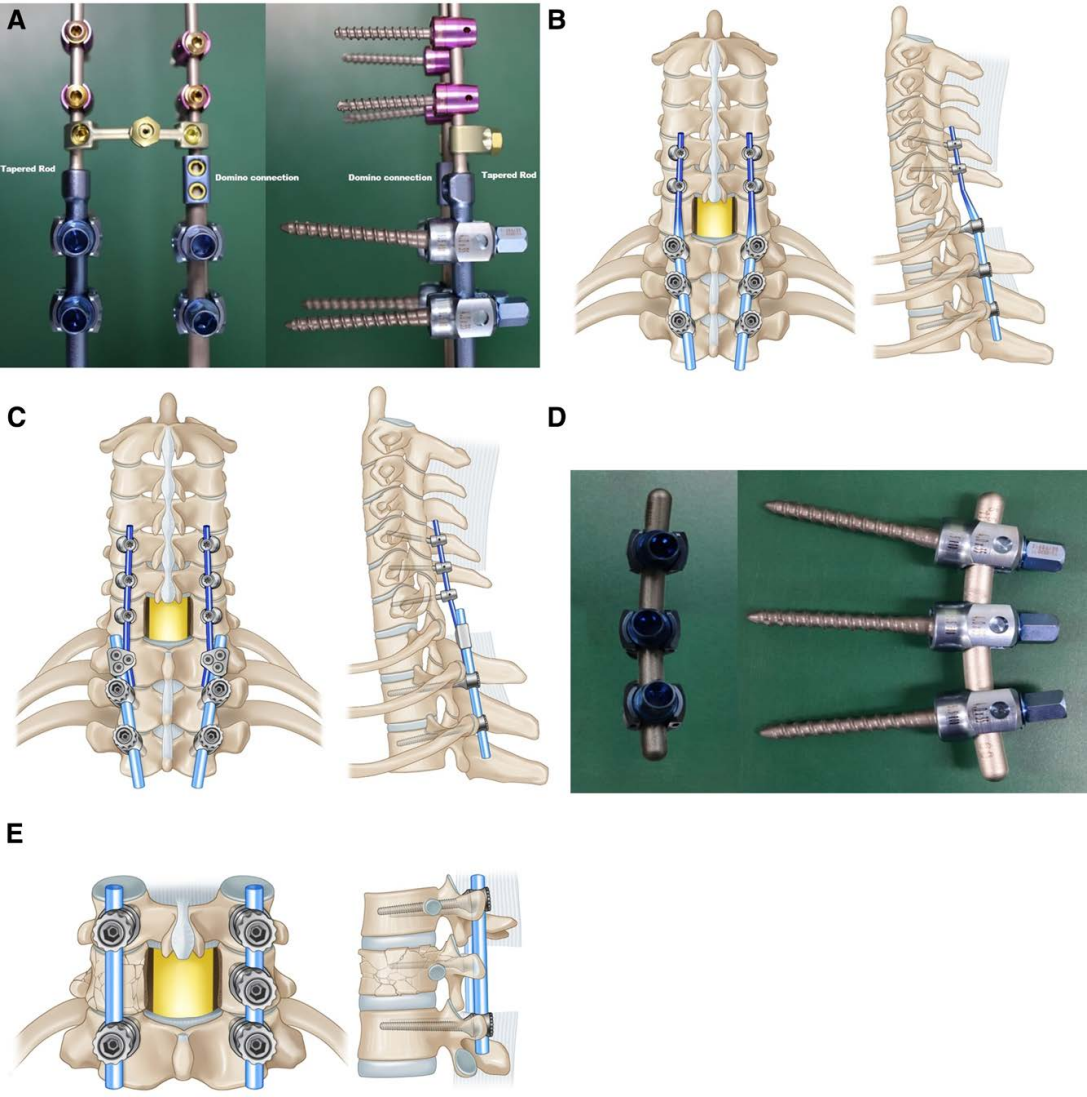
Three different types of rod connection for crossing cervical thoracic junction (CTJ). (A) A tapered diameter (3.5 and 5.5 mm) rod and 2 different diameter (3.5 and 5.5 mm) rods connected with Domino connection system, (B) screws from C5 vertebra to T3 vertebra were connected with a tapered diameter (3.5 and 5.5 mm) rod skipping the space-occupying screw on C7 vertebra, (C) screws from C5 vertebra to T3 vertebra were connected with 2 different diameter (3.5 and 5.5 mm) rods and Domino connection system skipping the space-occupying screw on T1 vertebra, (D) and (E) A 5.5-mm single-diameter rod to connect CPS without the need to skip the screw due to space occupation by the connecting system. Thus, this efficient connection using 5.5-mm single-diameter rod can give us an opportunity to reduce the maximal motion segments and operation time.

Different rod connection methods have been previously used, including single-diameter rods, accessory additional rods, and transitional diameter rods.^[3,6,7]^ However, there is no definite evidence for the ideal use of these 3 rod connection methods for the CTJ (Fig. 1).

Several previous studies have shown the efficacy and safety of cervical pedicle screw placement (CPS) through the posterior approach.^[8]^ We have previously presented the accuracy and safety of CPS for treating several cases with challenging spinal pathologies.^[9–15]^ Additionally, we previously demonstrated the efficacy of CPS use in combination with 5.5-mm single-diameter rods for treating metastatic spinal tumors.^[16,17]^ Therefore, this study aimed to demonstrate the efficacy of CPS combined with 5.5-mm single-diameter rods for treating various diseases in the CTJ.

## 2. Methods

This study was approved by the institutional review board (IRB) of our hospital (IRB No. 2023-1135). Informed consent was waived by the IRB due to the retrospective nature of this study.

### 2.1. Materials

Between March 2012 and December 2022, a total of 42 patients (male, n = 25; female, n = 17) underwent posterior cervical spinal surgeries that crossed the CTJ, with or without an anterior approach, performed by a single surgeon. The mean patient age was 58.6 years (range, 16–81 years). In all the included cohorts, only the Legacy^®^ pedicle screw system (Medtronic Sofamor Danek, Inc., Memphis) was used for the placement of pedicle screws on the cervical and thoracic vertebrae. C2 was the highest instrumented cervical vertebra, and T4 was the lowest instrumented thoracic vertebra.

In 5 categories of various CTJ diseases, 5.5-mm single-diameter rods were used for easy rod connection and to achieve biomechanical superiority, which resulted in relatively short-segment fixations. These results are presented by disease category.

### 2.2. Radiologic outcomes

We evaluated fusion by measuring the change in the interspinous distance of <1 mm between flexion and extension X-ray images. We measured the segmental Cobb angle loss between the immediate postoperative and final follow-up segmental Cobb angle. The segmental Cobb angle was defined as the angle between the upper endplate of the uppermost instrumented vertebra and the lower endplate of the lowest instrumented vertebra.

### 2.3. Surgical methods

The patients were placed in a prone position to allow the lamina plane to be horizontal and parallel to the ground. Motor-evoked potentials were monitored throughout the procedure, except in emergent conditions. The entry point of the screw was determined from the sagittal and axial computed tomography images, and it was defined as the notch level in the sagittal plane and medial to the lateral border of the superior articular process by 1-quarter of its width in the axial plane (or by 1-half of its width at C7).^[9]^ A small pilot hole was made at the predetermined entry point with a 1.8-mm diameter match head-type burr. A small, curved pedicle probe (2.5 mm in diameter) was slowly inserted vertically into the global lamina plane with a medial trajectory through the cortical hole, and the tip was placed at the thick medial cortical pedicle wall. Upon locating the cancellous channel, the medially directed force of the probe led to an insertion depth of approximately 30 mm. After forming a track with the curved probe, ball tip probe palpation was performed. Then, a straight pedicle probe, tapping, and screw were inserted. The aforementioned procedures were performed using a freehand technique or under navigation guidance, depending on the availability of the equipment. A detailed technical description of CPS was previously described.^[9,10,12,13,18]^ After screw insertion, appropriate decompression or reduction was performed. Acceptable instrumentation position and alignment were identified using intraoperative X-ray and computed tomography images, if available.^[18]^

## 3. Results

### 3.1. Trauma

**Figure 2.**
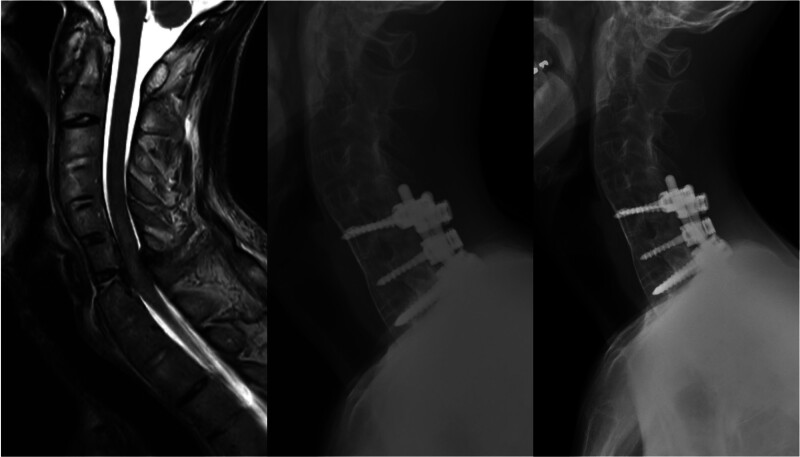
A 60-yr-old male patient with a burst fracture and cord compression at the C6–C7 level. Fusion was made from C6 to T1, and complete fusion was observed on flexion and extension images obtained 1-yr postoperatively.

Of the 4 patients with trauma (mean age, 60.5 years; range, 39–75 years), 3 were male and 1 was female. Three patients had facet dislocations or subluxations, and 1 had a burst fracture with ankylosing spondylitis. The screws were placed from C6 to T1 in 3 patients and from C6 to T2 in 1 patient, using CPS and 5.5-mm single-diameter rods for 3 patients (Fig. 2).

One complication occurred, and a C7–T1 fixation was placed on the 39-year-old patient; however, this further worsened the subluxation (i.e., spondyloptosis) 1 month after the surgery. The patient underwent revision by reducing and fixating the C6–T2 again, and this case was previously reported.^[19]^ We achieved complete fusion, and the American Spinal Injury Association Impairment Scale (AIS) improved from AIS D to AIS E in all 4 patients.

### 3.2. Dumbbell tumor on C7–T1

**Figure 3.**
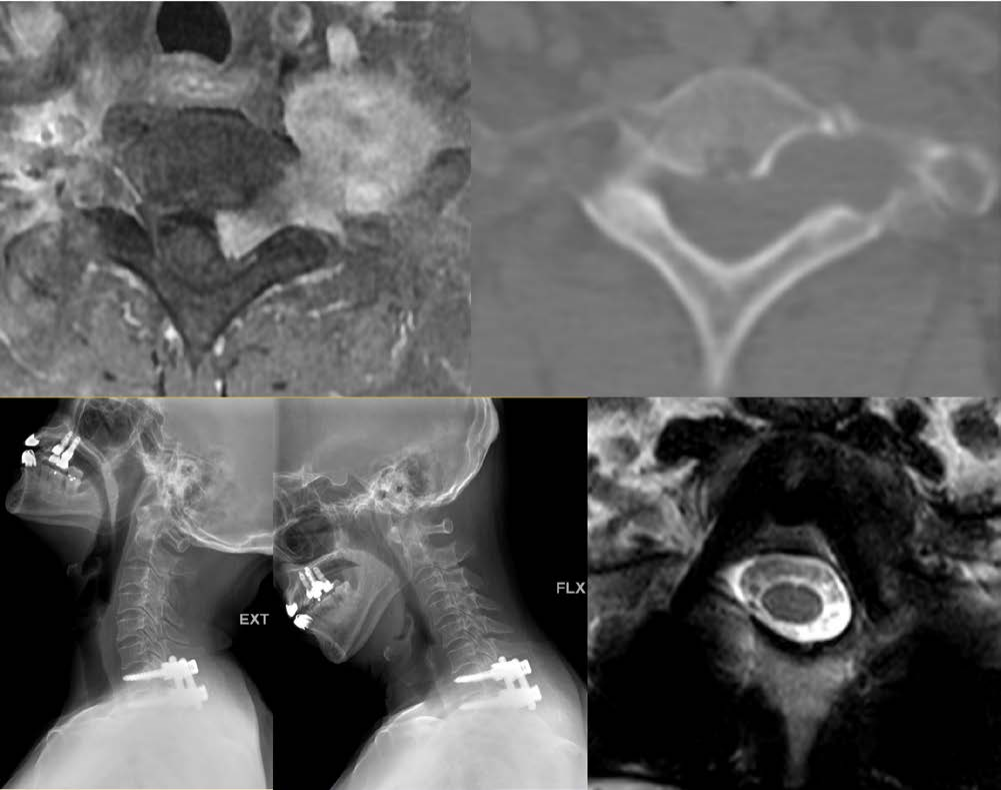
A 60-yr-old female patient with a dumbbell schwannoma on C7–T1, Lt., underwent total tumor resection and C7–T1 fusion. Postoperative 3-year dynamic X-ray imaging revealed complete fusion.

In a 60-year-old female patient with dumbbell schwannoma (Fig. 3) and a 16-year-old male patient with peripheral nerve sheath tumor, CPS with 5.5-mm single-diameter rods was performed at the C7–T1 level, and removal of the tumor was performed through the unilateral facet joint. Despite the absence of a pedicle because of tumor erosion on the unilateral C7 vertebra, the screw was placed on the body of C7 directly through the virtual pedicle tracts (Fig. 3). To achieve short-segment fusion in the biomechanically susceptible CTJ with unilateral absence of the C7 pedicle, 5.5-mm single-diameter rods were used. Fusion was achieved in the 60-year-old patient with schwannoma; however, the 16-year-old patient with a malignant tumor died from tumor metastasis 1 year after surgery.

### 3.3. Deformity (instrument loosening and flat neck)

**Figure 4.**
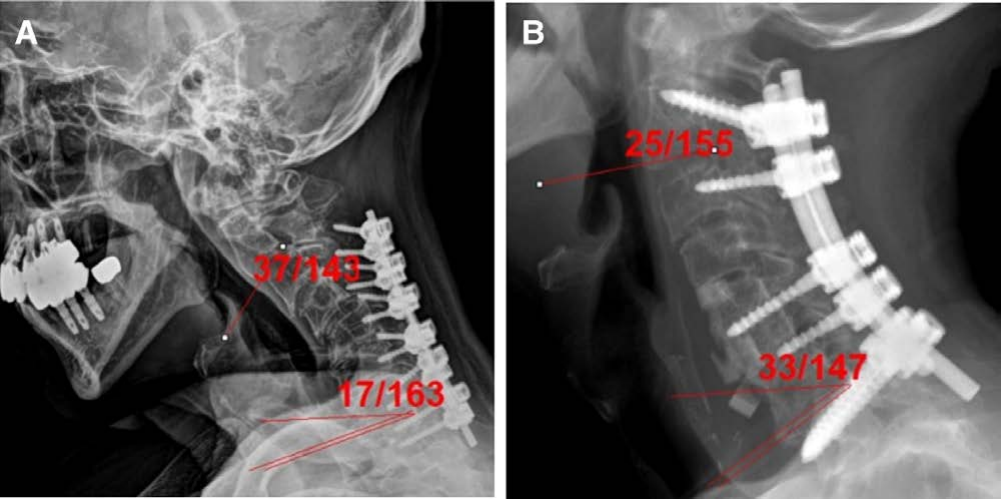
A 64-yr-old male patient complained of neck pain and difficulty raising his head. (A) A simple lateral X-ray image showed pulled-out screws and global kyphosis. Anterior and posterior approaches were performed, as well as C7 pedicle subtraction osteotomy. (B) Postoperative 1-year X-ray lateral image showed a well-balanced spine and complete fusion.

A 64-year-old male patient had a cervical spinal fusion at a different hospital. Although the lateral mass and pedicle screws were placed from C3 to T1, screw pullout, nonunion, and kyphosis developed. The patient’s neck pain was severe (numeric rating scale: 9), and they could not raise their head straight. The C2–C7 sagittal vertical axis was >70 mm, and the T1 slope was 17°; however, the cervical lordosis was − 37° kyphosis on the standing lateral X-ray image (Fig. 4A).

Correction surgery involving pedicle subtraction osteotomy on C7 was performed, with fixation from C2 to T1 using CPS with 5.5-mm single-diameter rods, and complete fusion was identified on the lateral X-ray image 1-year postoperatively. The C2–C7 sagittal vertical axis was 45 mm, the T1 slope was 33°, and cervical lordosis was 22° lordosis on the standing lateral X-ray image (Fig. 4B).

### 3.4. Metastatic spinal tumors

Between March 2012 and December 2022, 31 consecutive patients with metastatic cervicothoracic junctional tumors (male, n = 17; female, n = 14; mean age, 59.6 years; range, 24–77 years) underwent posterior cervical spinal fusion surgery, performed by a single surgeon.

Palliative spinal reconstruction is performed using CPS for metastatic spinal lesions when the life expectancy is greater than 3 months, followed by adjuvant radiotherapy. Metastatic tumors compressing the spinal cord from C7 to T2 were included in this study. All surgeries, including decompression and stabilization using CPS crossing the CTJ, were conducted through the posterior approach, except in 6 cases.

**Figure 5.**
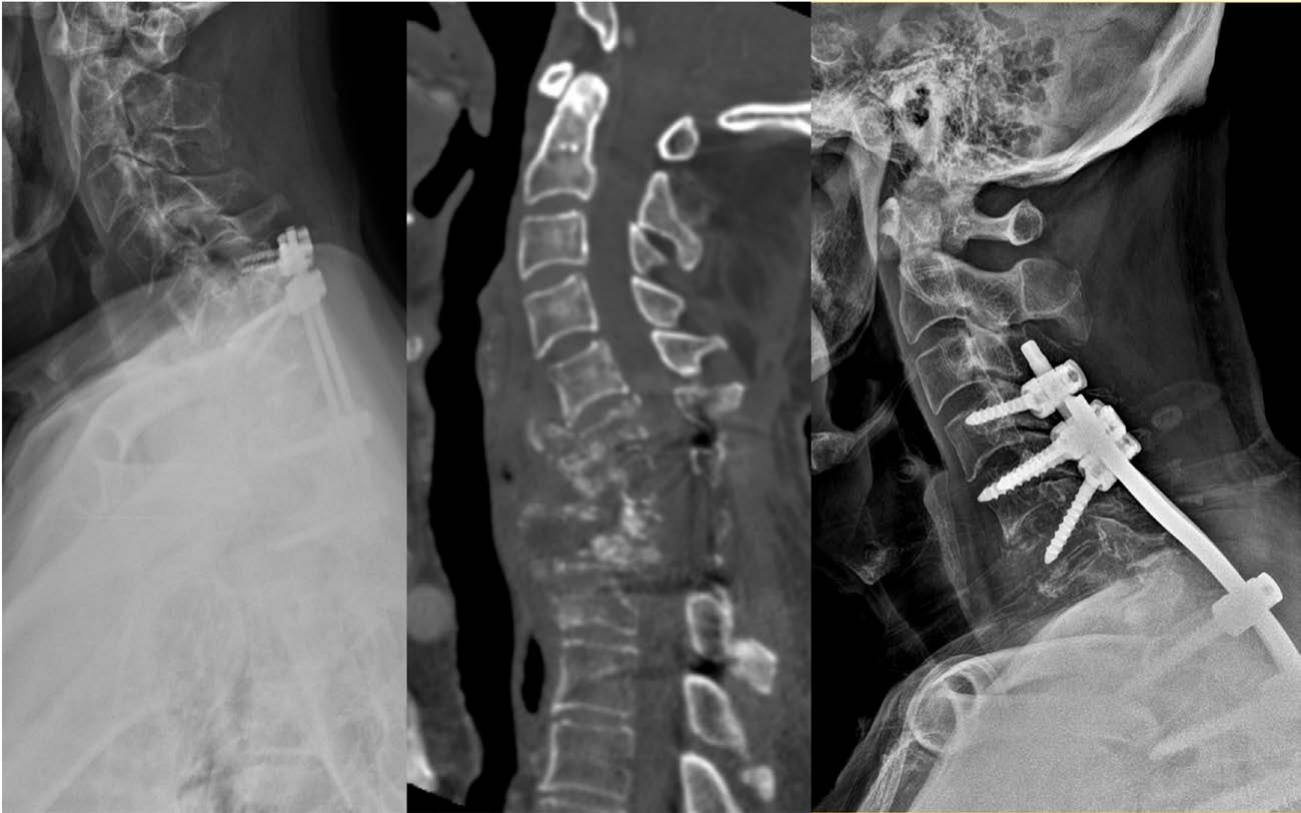
A 65-yr-old male patient with pancreatic cancer had instrument failure and metastatic tumor progression resulting in CTJ kyphosis. Revision was successful with CPS and 5.5-mm single-diameter rods.

Anterior cages filled with allograft bone chips were inserted in 6 patients. Three cases of revision surgery were performed, in which the primary surgery was performed at a different hospital, after which tumor recurrence and instrumentation failure developed (Fig. 5 and Table 1).

**Table 1:** Demographics and clinical outcomes of patients with metastatic cervicothoracic junctional tumors and clinical outcomes.

	Total (n = 31)
Age (year, mean ± SD, [range])	59.65 ± 13.10 (24–77)
Sex (*n*, %)	
Male	17 (54.8%)
Female	14 (45.2%)
Histology (*n*, %)	
Lung	7 (22.6%)
Pancreas	6 (19.4%)
Liver	4 (12.9%)
Renal	4 (12.9%)
Breast	2 (6.5%)
Colon	2 (6.5%)
Others*	6 (19.4%)
Revision (*n*, %)	
Yes	3 (9.7%)
No	28 (90.3%)
Anterior support (*n*, %)	
Yes	6 (19.4%)
No	25 (80.6%)
Preoperative SINS (mean ± SD)	14.10 ± 1.78
Neck NRS (mean ± SD)	
Preoperative	7.7 ± 2.7
Postoperative	1.1 ± 1.5
SOSGOQ	
Preoperative (mean ± SD)	18.4 ± 13.3
Postoperative (mean ± SD)	71.3 ± 16.6

NRS = numeric rating scale, SD = standard deviation, SINS = spinal instability neoplastic score, SOSGOQ = Spine Oncology Study Group Outcomes Questionnaire.

* Other primary cancer diagnosis included urothelial carcinoma, cardiac tumor, gastrointestinal stromal tumor, multiple myeloma, stomach cancer, and metastasis of unknown origin.

The mean numeric rating scale for neck pain and functional outcomes, as measured by the Spine Oncology Study Group Outcomes Questionnaire, improved after surgery (Table 1).

One revision surgery was performed for a deep wound infection, and 3 patients had incidental dural tears, which were primarily repaired during the operation. No screw or rod fractures or screw pullout occurred, and the segmental Cobb angle was relatively well preserved (Table 2).

**Table 2:** Surgical complications.

	(n = 31)
Postoperative revision surgery	
Yes	1 (3.2)
No	30 (96.8)
Complications	
Yes	4 (12.9)
Major*	1
Minor†	3
No	27 (87.1)
Instrument pullout or fracture	
Yes	0 (0)
No	31 (100)
Segmental cobb angle loss > 10°‡	
Yes	0 (0)
No	31 (100)

* Major complications included deep infections.

† Minor complications included dura tears.

‡ Segmental cobb angle loss = immediate postoperative – final follow-up segmental cobb angle.

### 3.5. Primary bone tumors

**Figure 6.**
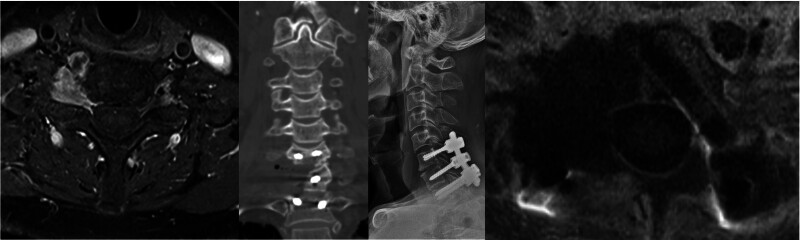
A 61-yr-old man with a C6–C7 right-side foraminal chondrosarcoma underwent half C7 vertebral spondylectomy and C6–C7–T1 fusion with posterior facet fusion.

Four patients underwent CTJ fusion and tumor removal surgery. All surgeries were performed after pathology confirmation through a percutaneous biopsy. Table 3 presents the details of the patients; among the 4 patients, cases 1 and 2 have been previously published.^[20]^ Half vertebrae spondylectomy was possible when the tumor had a lopsided location (Fig. 6).

**Table 3:** Primary bone tumor in the cervical thoracic junction.

No.	Sex	Age	Diagnosis	Level	Surgery	Fusion	F/U (y)	Tumor control/fusion
1	M	29	Giant cell tumor	C7	Total en bloc spondylectomy	C6–T1–T2 fusion with sternal bone graft	4	Yes/yes
2	M	49	Chordoma	C7	Total en bloc spondylectomy	C6–T1–T2 fusion with sternal bone graft	3	Yes/yes
3	M	61	Chodorosarcoma	C7	Half vertebra en bloc spondylectomy	C6–T1 (unilateral)–T3 with facet joint fusion	1	Yes/yes
4	F	81	Enchondroma	T1	Gross total resection with piecemeal fashion	C6–T1 (unilateral)–T3 with facet joint fusion	3	Yes/yes

## 4. Discussion

The CTJ is a biomechanically challenging region for spinal surgeries and one of the major risk factors for poor surgical outcomes.^[1–4,7]^ Traditionally, because of the different anatomical sizes of the cervical and thoracic spinal pedicles, different diameter rods were connected. However, these rod connections have 3 major drawbacks. First, the space-occupying connection instruments, such as a domino system, prohibit pedicle screw placement at the C7 or T1 level. This has inevitably led to screw placement above or below the instrumented levels (Fig. 1). Most surgeons implement lateral mass screw placement (LMS), which is connected with a thoracic spinal screw system using a domino system or tapered rods. However, to avoid instrumentation failure, LMS needs more fixation levels than CPS. Second, the connection of 2 rods with different diameters results in lower biomechanical stability compared with a single-diameter continuous rod, although some disagreements exist.^[3,21–23]^ Third, the procedure to connect 2 different diameter rods has longer operative times and larger blood loss volumes.

Previously, we introduced the safety and efficacy of CPS for treating several challenging cervical spinal diseases, including CTJ trauma and tumors, with a freehand technique.^[9,10,12,13,15,18,20,24]^ Because we got used to the freehand technique after a safe learning curve period, avoiding mismatched confusion between the navigation-guided images and the real rotated vertebral body has been helpful for us when we could use a navigation system during CPS.^[12,18]^ Although these advanced imaging technologies have improved the accuracy of CPS, they have also increased the costs and operative times, making the operating room cumbersome. As a result, surgeons relying on these technologies may lose surgical techniques and experience.^[9]^ In addition, because surgery for traumas and metastatic spinal tumors is frequently performed in emergency situations, our freehand technique based on CPS experience is extremely beneficial. A study described a navigation system as an essential tool for spinal surgery in the CTJ. However, this study also recommended that spine surgeons should not attempt instrument placement under navigation guidance if they have not performed the same procedure without navigation in this region.^[3]^

Traditionally, the instrument is placed at least 2 levels above and below the treatment site of the trauma or tumor in the CTJ.^[25,26]^ However, technological developments, including instruments and navigation systems, have helped reduce surgical complications and fuse only 2 segments, even in the CTJ. This means that 5.5-mm single-diameter rods can offer biomechanical superiority and be advantageous for short-segment fusions even in this biomechanically unstable area.

With these paradigm shifts, we have struggled to reduce the instrumentation levels and complications associated with long incisions when performing short-segment fusions, even for CTJ pathologies, which has resulted in our routine practice.^[18,25–27]^ For the biomechanical compensation of short-level instrumentations, we used the Legacy^®^ or Solera^®^ screw system (Medtronic Sofamor Danek, Inc., Memphis) with a maximal diameter and length that correspond to the vertebral pedicle size.

In the entire cohort, nearly 80% of patients had a fixation of <4 levels, compared with a previous study.^[7]^ Longer instrumentation (> 7 levels) was highly related to instrumentation failure.^[25]^ Our surgical policy for this region is always minimally short-segment fixation with decompression, and the single surgeon policy has been previously published.^[16,17,24]^

Interestingly, in the study cohort, no degenerative diseases were found for the proper use of 5.5-mm single-diameter rods, although we agreed to extend the instrumentation to the proximal thoracic spinal level when performing long-level posterior cervical spinal fusion surgery.^[28,29]^ When we connect the screws from the cervical spine to the upper thoracic spine for degenerative conditions, such as long-segment ossification of the posterior longitudinal ligament, we used the Vertex® system (Medtronic Sofamor Danek, Inc., Memphis) with 3.5-mm single-diameter rods.^[14,29]^ This indicates that a biomechanically relatively stable degenerative spine would not need 5.5-mm single-diameter rods.

This study has several limitations. This was a retrospective analysis with a small number of patients, and no comparison group was employed.

## 5. Conclusion

CPS combined with 5.5-mm single-diameter rods provides biomechanical stability for the treatment of challenging spinal diseases, such as trauma, benign or malignant tumors, or deformities in the CTJ. This technique allows for easy connection of the same rods and the opportunity to fuse short segments, as well as a short operation time and less blood loss.

## Author contributions

**Data curation:** Subum Lee.

**Formal analysis:** Sang Hyub Lee.

**Investigation:** Younggyu Oh, Sun Woo Jang.

**Resources:** Danbi Park.

**Supervision:** Jin Hoon Park.

**Visualization:** Chongman Kim.

**Writing – original draft:** Younggyu Oh.
